# Comprehensive comparison on the anti-inflammation and GC-MS-based metabolomics discrimination between *Bupleuri chinense* DC. and *B*. *scorzonerifolium* Willd

**DOI:** 10.3389/fphar.2022.1005011

**Published:** 2022-09-14

**Authors:** Mingming Zhao, Linxuan Xiao, Ke-Gang Linghu, Guanding Zhao, Qiling Chen, Liyu Shen, Parsa Dar, Meiwan Chen, Yuan Hu, Jinming Zhang, Hua Yu

**Affiliations:** ^1^ State Key Laboratory of Southwestern Chinese Medicine Resources, Chengdu University of Traditional Chinese Medicine, Chengdu, China; ^2^ Institute of Chinese Medical Sciences, State Key Laboratory of Quality Research in Chinese Medicine, University of Macau, Macao, Macao SAR, China; ^3^ Macao Centre for Research and Development in Chinese Medicine, Institute of Chinese Medical Sciences, University of Macau, Macao, Macao SAR, China

**Keywords:** Bupleuri radix, anti-infammation, metabolomics, GC-MS, species discrimination

## Abstract

Bupleuri Radix (BR) is a traditional Chinese medicine and widely used for cold and fever, influenza, inflammation, hepatitis and menstrual diseases. Two authentic medicinal plants of *Bupleuri chinense* DC. (Beichaihu, BCH) and *B. scorzonerifolium* Willd. (Nanchiahu, NCH) are recommended by the current Chinese Pharmacopoeia for BR. In the present study, the comparative investigations on the anti-inflammatory effects and gas chromatography-mass spectrometry (GC-MS)-based metabolomics for the species discrimination of BCH and NCH were conducted and reported. The *in vitro* evaluations indicated that the supercritical fluid extracts (SFEs) (IC_50_ of 6.39 ± 0.52 and 1.32 ± 0.05 mg (herb)/mL for BCH and NCH) were determined to be more potent than those of the hydro-distillation extracts (HDEs) (IC_50_ of 203.90 ± 8.08 and 32.32 ± 2.27 mg (herb)/mL for BCH and NCH) against LPS-induced inflammation in RAW264.7 macrophages. The higher anti-inflammatory effects of NCH were associated to its different chemical compositions to the BCH as characterized by the GC-MS analysis. Furthermore, based on the metabolomics and deep chemometric approaches, a minimum combination containing 15 chemical markers was optimized from the identified components and successfully applied for the species discrimination of BCH and NCH. This study not only helps to comparative understand BCH and NCH both in phytochemistry and pharmacology, but also provides the potential chemical markers for improvement of methods for the quality control of BCH and NCH.

## Highlights

• The SFEs exhibited better anti-inflammatory activity than the HDEs for both BCH and NCH.
• NCH presented higher potency on anti-inflammation than BCH.
• BCH and NCH showed significant difference in chemical compositions.
• A combination of 15 components was successfully discovered from the GC-MS-based metabolomics approach for the species discrimination of BCH and NCH.

## Introduction

**FIGURE 1:**
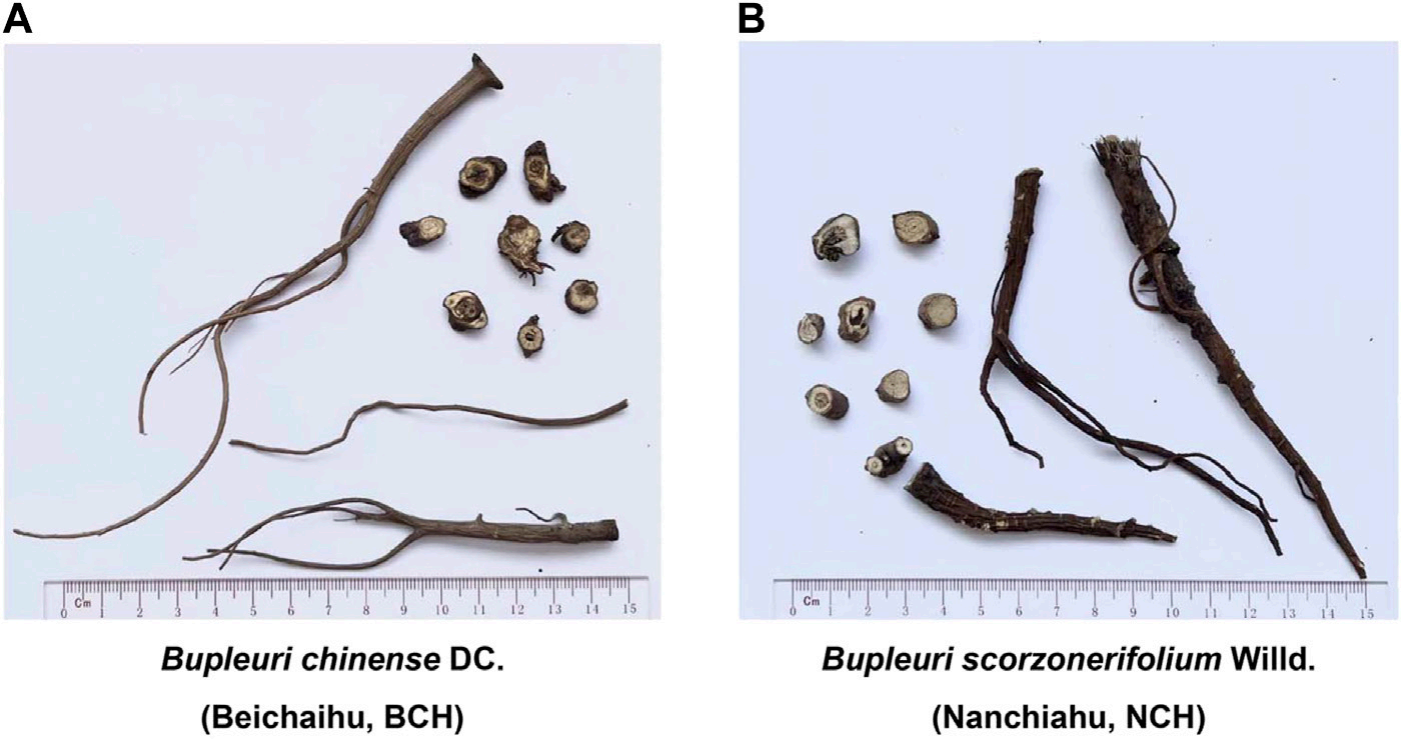
The herbal materials of *Bupleuri chinense* DC. (Beichaihu, BCH) **(A)** and *Bupleuri scorzonerifolium* Willd. (Nanchiahu, NCH) **(B)**.

Bupleuri Radix (BR, also named as Chaihu) is a traditional Chinese medicine and has been widely used in China for over 2000 years for its broad pharmacological activities and human health benefits (Yuan et al., 2017). Until now, the known plants of Bupleurum genus in China include 36 species, 17 varieties and seven variants, and 20 of which were/are practically used as BR in clinical applications (Yang et al., 2017; Sun et al., 2019). Although most of these plants vary slightly in morphologic appearance, their appreciable difference in chemicals and bioactivities attracts increasing attention. For example, the plant of *Bupleuri longiradiatum* Turcz has been forbidden to be used as BR by the Chinese pharmacopeia in clinics due to its significant toxicities (Yang et al., 2017). Currently, only two species of *B. chinense* DC. (also called Beichaihu, BCH) (Figure 1A) and *B. scorzonerifolium* Willd. (also called Nanchaihu, NCH). (Figure 1B) are recommended by the Chinese pharmacopeia (2020 version) to serve as the plant sources for BR.

In clinical applications, BR has been widely used for treatment of fever, pain, and inflammation associated with influenza or the common cold (Yang et al., 2017). The modern pharmacological investigations reported that BR presented broad biological activities including anti-influenza (Yan et al., 2022), anti-tumor (Zhao et al., 2019; Zhou et al., 2021), anti-inflammation (Jiang et al., 2020), anti-depression (Guo et al., 2020; Chen et al., 2021), anti-stress (Wang P. et al., 2021; Wang Z. et al., 2021)and antipyretic (Wang et al., 2019), antiviral (Du et al., 2018), antimicrobial (Liu et al., 2016), immunomodulatory (Tang et al., 2021) and hepatoprotective effects (Ren et al., 2019). Although different types of chemicals have been reported to contain in BCH (saponins, volatile oils, flavonoids and polysaccharides) (Xing et al., 2015) and NCH (saponins, lignans, fatty acids, volatile oils and polysaccharides) (Jiang et al., 2020), the saponins and volatile oils have been investigated to be the most important parts for both BR species which are associated to their antipyretic (Xing et al., 2015; Li et al., 2018) and anti-inflammatory effects (Kim et al., 2015; Ma et al., 2016). The pharmaceutical products (BR injection, major BR decoction and minor BR decoction) containing such ingredients have been well developed and widely used in clinics (Ni et al., 2009; Chen et al., 2010).

Phytochemically, BCH was reported to mainly contain saponins, volatile oils, flavonoids and polysaccharides (Xing et al., 2015); while NCH was reported to be rich in saponins, lignans, fatty acids, volatile oils and polysaccharides (Jiang et al., 2020). The chemical difference between them could also be mentioned as their different description as aroma smelling for BCH and transmutative-oil-like smelling for NCH under the item of ‘Properties’ by the Chinese Pharmacopeia. All these evidences suggested the significant difference between BCH and NCH in chemistry despite both of them are used as BR in clinical applications. Nevertheless, up to date, the comparative investigation for BCH and NCH is still not reported.

In this study, we aim to provide a comparative comprehension on BCH and NCH in order to improve their quality control for product development and clinical applications. The anti-inflammatory effects were compared to find out the active extracts from these two herbs. Furthermore, a chemical combination with minimum numbers of components was optimized for the species discrimination of BCH and NCH using the GC-MS-based metabolomics and deep chemometric analyses. This study not only helps to comparatively understand BCH and NCH both in phytochemistry and pharmacology, but also provides the potential chemical markers for the further quality control of BR herbs.

## Materials and methods

### Chemicals

Dimethyl sulfoxide (DMSO), lipopolysaccharides (LPS) from *Escherichia coli* O111:B4 and 3-[4,5-Dimethyl-2-thiazolyl]-2,5-diphenyltetrazolium bromide (MTT) were purchased from Sigma-Aldrich (St. Louis, MO, United States). Methanol of HPLC grade were purchased from Sigma-Aldrich (St. Louis, MO, United States). Ethanol (HPLC grade) was purchased form RCI Labscan Limited (Thailand). All other reagents and chemicals were of analytical grade. Milli-Q water was prepared using a Milli-Q system (Millipore, MA, United States).

### Herbal materials

Total 18 batches of the BR samples including 10 batches of *B. chinense* DC (BCH) and eight batches of *B. scorzonerifolium* Willd (NCH) were collected from different regions in China (Table 1). All samples were authenticated by the corresponding authors and the voucher specimens were deposited at the Institute of Chinese Medical Sciences, University of Macau, Macao, China. Prior to experiments, the herbal materials were dried, powdered and passed through 65 mesh sieve.

**TABLE 1:** Information of collected BR samples.

Batch no.	Species	Location	Collecting time (year)
BCH001	*B. chinense* DC	Kangle County, Lanzhou City, Gansu Province	2019
BCH002	*B. chinense* DC	Kangle County, Lanzhou City, Gansu Province	2019
BCH003	*B. chinense* DC	Jishan County, Yuncheng, Shanxi Province	2019
BCH004	*B. chinense* DC	Wenxi County, Yuncheng City, Shanxi Province	2019
BCH005	*B. chinense* DC	Wenxi County, Yuncheng City, Shanxi Province	2020
BCH006	*B. chinense* DC	Zhangjiakou, Chengde, Hebei Province	2019
BCH007	*B. chinense* DC	Zhangqiu, Jinan, Shandong Province	2019
BCH008	*B. chinense* DC	Shanxi Province	2019
BCH009	*B. chinense* DC	Flychi County, Sanmenxia, Henan Province	2019
BCH010	*B. chinense* DC	Xinjiang County, Shanxi Province	2020
NCH001	*B. scorzonerifolium* Willd	Longfeng District, Daqing City, Heilongjiang Province	2019
NCH002	*B. scorzonerifolium* Willd	Yuncheng City, Shanxi Province	2019
NCH003	*B. scorzonerifolium* Willd	Yuncheng City, Shanxi Province	2019
NCH004	*B. scorzonerifolium* Willd	Longfeng District, Daqing City, Heilongjiang Province	2020
NCH005	*B. scorzonerifolium* Willd	Dulbot County, Daqing City, Heilongjiang Province	2020
NCH006	*B. scorzonerifolium* Willd	Hinggan League, Holanhot, Inner Mongolia Province	2020
NCH007	*B. scorzonerifolium* Willd	Lindian County, Daqing City, Heilongjiang Province	2020
NCH008	*B. scorzonerifolium* Willd	Dulbot County, Daqing City, Heilongjiang Province	2020

### Herbal extraction

#### Hydro-distillation

Approximately 800 g of mixed powders of BCH (10 batches, 80 g for each) or NCH (8 batches, 100 g for each) were soaked with 8 L of distilled water for 3 h, and then extracted with the hydro-distillated method for another 12 h. The collected volatile oils (HDEs) of BCH (yield: 0.1%) and NCH (yield: 0.25%) were stored at -20°C in amber glass bottles for further experiments.

#### Supercritical fluid extraction

The SFE extraction of BCH and NCH was carried out on a supercritical fluid extractor coupled with fully automated pilot scale systems (Supercritical Fluid Technology, INC., United States, Model: SFT250). Briefly, 100 g powder of BCH or NCH was loaded into the cylindrical stainless-steel extraction tank and then extracted with CO_2_ (purity 99.5%) fluid under the following parameters: extraction kettle pressure of 260 Pa; vessel temperature of 60°C; vessel oven temperature of 70°C; and the extraction time of 2 h. Finally, the SFE extracts (SFEs) were collected in a 50 ml centrifuge tube (average yields of 1.16 ± 0.06% and 3.57 ± 0.44% for BCH and NCH, respectively), and stored at −20°C for further experiments.

### Comparison of anti-inflammatory activities between BCH and NCH

#### Cell culture and cytotoxicity

RAW264.7 cells were obtained from the American Type Culture Collection (ATCC; Manassas, VA, United States). The cells were cultured in Dulbecco׳s modified eagle medium (DMEM) supplemented with 10% heat-inactivated fetal bovine serum (FBS) and 1% penicillin/streptomycin (P/S) (ThermoFisher Scientific Inc., United States) at 37°C in humidified 5% CO_2_ atmosphere (Linghu et al., 2020a). When the cells reached 80% confluence, the cells were sub-cultured after scraping from a 25 cm^2^ flask.

The cytotoxicity of all extracts on RAW264.7 cells was assessed using the MTT assay (Linghu et al., 2020a). Briefly, RAW264.7 cells (1×10^4^ cells/well) were seeded in 96-well plates and allowed to adhere overnight. Prior to cell treatment, both HDEs and SFEs were completely dissolved in DMSO and diluted with culture medium for further cell treatment. The cells were treated with the indicated concentrations of HDEs or SFEs for 24 h, and then incubated for an additional 1 h with fresh culture medium containing 0.5 mg/ml MTT. Subsequently, the culture medium was removed and the absorbance of the dissolved precipitate (in 150 μL of DMSO) was measured at 490 nm using a microplate reader (FlexStation3; Molecular Devices, United States).

#### Anti-inflammatory effects

The anti-inflammatory effects of BCH and NCH extracts (HDEs and SFEs) were comparatively investigated by evaluating on the inhibition of nitric oxide (NO) production in LPS-induced RAW264.7 macrophages. Briefly, RAW264.7 cells (1×10^4^ cells/well) were seeded onto 96-well plates and allowed to adhere overnight. Subsequently, the cells were pre-treated with indicated concentrations of individual extract for 1 h, and then stimulated with LPS (1 μg/ml) for 24 h. NO production was determined by measuring the nitrite accumulated in the medium with Griess reagent.

### Discrimination of BCH and NCH with GC-MS-based metabolomics approach

#### GC-MS analysis

The chemical profiles of the SFE extracts from BCH and NCH were characterized using a Thermo Trace 1,300 gas-chromatography (GC) instrument equipped with a thermo ISQ LT single quadrupole mass spectrometer, a TRIPLUS RSH autosampler for liquid, static headspace and solid phase microextraction injections, a split/splitless injector and a Xcalibur chromatography processing system (Thermo Fisher, United States). Sample separation was preformed using an DB-5MS (30 m × 0.25 mm, 0.25 μm film thickness) capillary column (Agilent, United States). The electron-impact (EI) mode was selected and the ionization voltage was 70 eV. Both the interface and ionization source temperatures were 280°C. Mass spectral scan range was set at 50–550 m*/z* with a scan rate of 0.60 s per scan. The oven temperature programming was set as follows: 1 min at 140°C, 5°C/min to 180°C, holding for 2 min; 3°C/min to 280°C, holding for 2 min; 30°C/min to 300°C, holding for 1 min. The samples were injected in 30:1 split mode, and the injection temperature and volume were 280°C and 2 μL, respectively.

#### Data processing and markers identification

The raw data of SFE extracts for BCH and NCH from GC-MS analysis was extracted and introduced to Progenesis QI software (version 2.0, Waters Corporation, MA, United States) for data processing, including peak detection, alignment and normalization. The compounds were identified by comparing the mass spectra of the detected components in GC-MS with those records in National Institute of Standards and Technology (NIST) mass spectral library and linear retention indices (LRI) calculated relative to (C8-C20) n-alkanes with LRI database using the NIST Mass Spectral Library. To further evaluate comprehensively quality and explore differential markers between BCH and NCH, the processed data including peak intensity, m/z and retention time of identified compounds was analyzed by a series of deep chemometric analysis including supervised regularized canonical correlation analysis (SRCCA), hierarchical clustering analysis (HCA) and partial least squares discriminant analysis (PLS-DA) using the Metaboanalyst website (https://www.metaboanalyst.ca).

### Statistical analysis

Data were analyzed using GraphPad Prism 6.0 software. All data are presented as mean ± S.D., and each experiment was performed at least three times. Significant differences between groups were determined using one-way ANOVA with Dunnet’s multiple comparisons test or unpaired *t*-test. *p* < 0.05 was considered difference significantly.

## Results

### Anti-inflammatory activities between HDEs and SFEs of BCH and NCH

Excessive NO release is one of important indexes for activated macrophages to inflammatory response. The capability on inhibition of NO production could be used to assess the anti-inflammatory effects of drug candidates (Linghu et al., 2020a; Linghu et al., 2020b).

**FIGURE 2:**
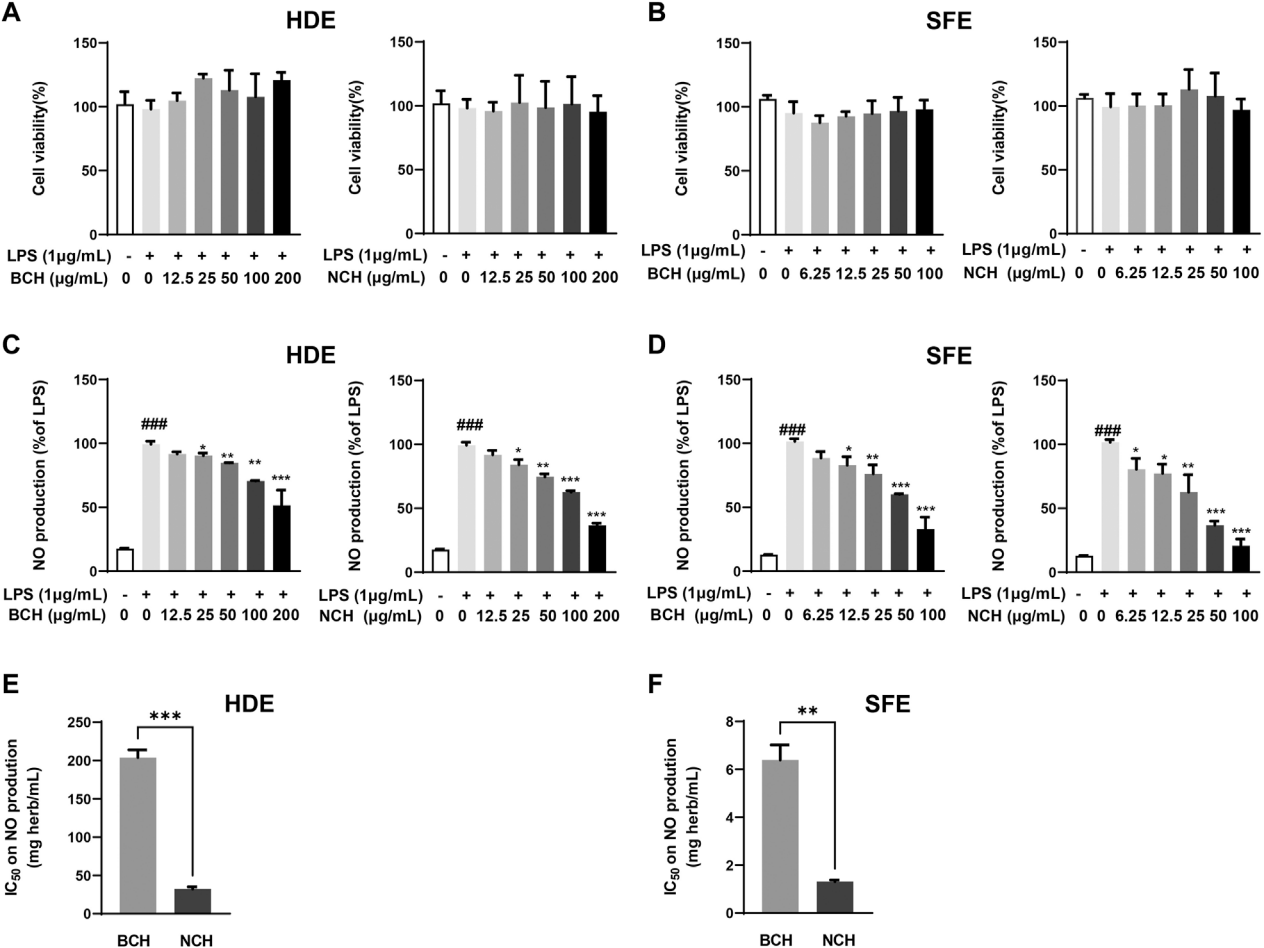
The anti-inflammation activity of HDE and SFE from BCH and NCH on RAW264.7 cells. **(A)** Cytotoxicity of HDE from BCH and NCH on RAW264.7 cells; **(B)** cytotoxicity of SFE from BCH and NCH on RAW264.7 cells; **(C)** anti-inflammatory effects of HDE from BCH and NCH through the measurement of accumulated nitrite; **(D)** anti-inflammatory effects of SFE from BCH and NCH through the measurement of accumulated nitrite; **(E)** IC_50_ of HDE from BCH and NCH on NO production in RAW264.7 cells; **(F)** IC_50_ of SFE from BCH and NCH on NO production in RAW264.7 cells. *p < 0.05, **p < 0.01, ***p < 0.001 versus LPS group. ^###^ *p* < 0.001 versus control group. Data are presented as mean ± S.D. (*n* = 3).

The cytotoxicity of HDEs and SFEs for BCH and NCH were illustrated in Figure 2 A and B. Under the nontoxic concentrations, both HDEs and SFEs of BCH and NCH presented the dose-dependent inhibition on NO production in LPS-induced RAW264.7 macrophages (Figures 2C,D). The average 50% inhibitory concentration (IC_50_) of the equaled herbal concentration for HDEs on NO production were determined to be 203.90 ± 8.08 and 32.32 ± 2.27 mg (herb)/mL for BCH and NCH (Figure 2E), as well as those of 6.39 ± 0.52 and 1.32 ± 0.05 mg (herb)/mL for the SFEs of BCH and NCH, respectively (Figure 2F). The results indicated the higher anti-inflammatory effects of SFEs than that of HDEs for BCH and NCH, as well as the better potency of NCH than that of BCH.

### Chemical characterization of SFEs from BCH and NCH

Based on the above *in vitro* comparison on LPS-induced RAW264.7 macrophages, the SFEs of both BCH and NCH were determined to be much potent than the HDEs on anti-inflammation. Therefore, the chemical compositions of the SFEs were further characterized by GC-MS analysis.

**FIGURE 3:**
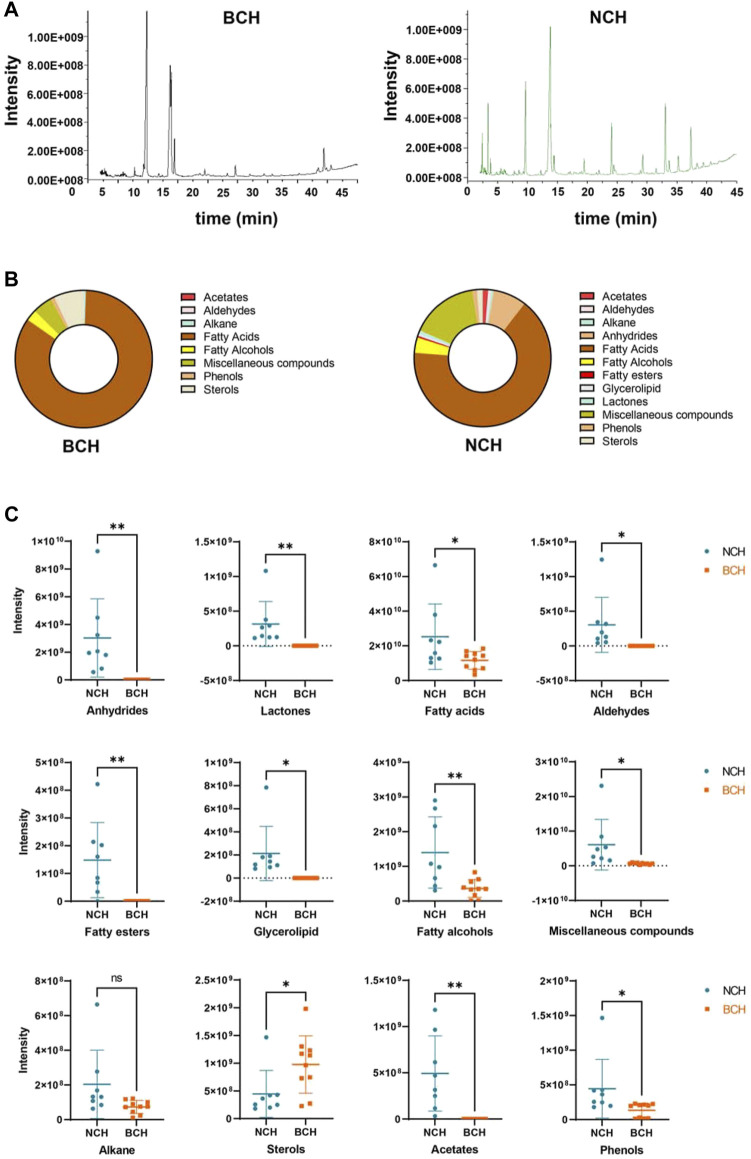
Chemical characterization of SFEs from BCH and NCH. **(A)** The typical total ion chromatograms (TIC) of the representative samples of BCH (BCH001) and NCH (NCH001) by GC-MS analysis; **(B)** the compositional features in SFEs from BCH and NCH; and **(C)** the characteristic distribution of different types of components in SFEs from BCH and NCH. Data are presented as Mean ± S.D. (*n* = 10 for BCH and *n* = 8 for NCH). *p < 0.05 and **p < 0.01 by unpaired *t*-test.

With the established GC-MS method, all SFE samples of BCH (10 batches) and NCH (8 batches) were chromatographically analyzed. The typical total ion chromatograms (TIC) of the representative samples for BCH (BCH001) and NCH (NCH001) were illustrated in Figure 3A. Furthermore, by comparing the mass spectra of the detected components in GC-MS with those records in National Institute of Standards and Technology (NIST) mass spectral library, as well as their retention indices with published literatures (Tykheev et al., 2018; Li et al., 2005), 28 components in BCHs (60.99–70.56% of the total peak area) and 44 components in NCHs (74.04–93.28% of the total peak area) were identified and summarized in Table 2. Among them, 24 components were found both in BCH and NCH, while 4 and 20 specie-specific components were found in BCH and NCH, respectively. The most abundant components in BCH were palmitic acid (25.4% on average), linoleic acid (15.8% on average), oleic Acid (6.1% on average) and octadecanoic acid (4.4% on average). And the most abundant components in NCH were linoleic acid (12.4% on average) and palmitic acid (8.8% on average), as well as other two specific components of mandenol (7.5% on average) and dodecanoic acid (4.7% on average). These results indicated the significant differences in chemical compositions between BCH and NCH both in quality and quantity.

**TABLE 2:** The information of the identified components in SFEs from BCH and NCH by GC-MS.

RT (min)	Formula	Name	Molecular weight	Ri	%Area																	
					1	2	3	4	5	6	7	8	9	10	11	12	13	14	15	16	17	18
2.22	C_13_H_28_	Tridecane	184.22	1,300	ND	ND	ND	ND	ND	ND	ND	ND	ND	ND	0.2	0.2	0.1	0.2	0.2	0.2	0.3	0.3
2.30	C_11_H_22_O_2_	Undecanoic acid	186.16	900	ND	ND	ND	ND	ND	ND	ND	ND	ND	ND	0.6	0.3	0.2	0.6	0.6	0.5	0.3	0.2
2.36	C_17_H_36_	2,6,10-Trimethyltetradecane	240.28	826	0.3	0.3	0.7	0.5	1.0	0.3	0.2	0.5	0.2	0.2	ND	ND	ND	ND	ND	ND	ND	ND
2.41	C_12_H_26_O	1-Dodecanol	186.20	925	ND	ND	ND	ND	ND	ND	ND	ND	ND	ND	3.1	1.5	0.7	3.8	6.7	2.7	1.9	1.7
2.61	C_15_H_32_	Pentadecane	212.25	923	0.2	0.2	0.1	0.3	0.1	0.1	0.2	0.1	0.3	0.1	0.1	0.1	0.1	0.3	0.2	0.2	0.2	0.2
2.69	C_11_H_24_O	1-Undecanol	172.18	877	ND	ND	ND	ND	ND	ND	ND	ND	ND	ND	0.3	ND	ND	0.9	ND	ND	ND	ND
2.71	C_14_H_22_O	2,4-Di-tert-butylphenol	206.17	1,519	1.0	0.8	0.9	1.1	1.0	0.7	0.1	0.1	0.0	0.2	0.4	0.6	0.7	ND	1.0	0.9	0.8	0.7
2.86	C_13_H_26_O_2_	Lauric acid, methyl ester	214.19	715	ND	ND	ND	ND	ND	ND	ND	ND	ND	ND	0.3	0.4	0.4	0.6	0.6	0.5	0.4	0.4
2.92	C_35_H_7_0	17-Pentatriacontene	490.55	781	0.4	0.3	0.3	0.5	0.3	0.2	0.1	0.1	0.1	0.2	0.4	ND	0.3	0.4	0.2	0.3	0.1	0.0
2.94	C_18_H_36_	trans-3-Octadecene	252.28	884	ND	ND	ND	ND	ND	ND	ND	ND	ND	ND	0.3	0.3	0.3	0.4	0.7	0.4	0.3	0.3
3.03	C_13_H_28_O	1-Tridecanol	200.21	825	0.4	0.3	0.4	0.5	0.3	0.2	0.1	0.1	0.0	0.1	ND	ND	0.2	ND	ND	ND	ND	ND
3.05	C_13_H_18_O_2_	Ibuprofen	206.13	838	ND	ND	ND	ND	ND	ND	ND	ND	ND	ND	0.3	0.3	0.2	0.3	0.3	0.2	0.2	0.2
3.39	C_12_H_24_O_2_	Dodecanoic acid	200.18	961	ND	ND	ND	ND	ND	ND	ND	ND	ND	ND	6.3	2.7	ND	5.4	6.8	5.0	4.1	2.7
3.81	C_14_H_28_O_2_	Lauryl acetate	228.21	902	ND	ND	ND	ND	ND	ND	ND	ND	ND	ND	1.1	0.4	0.1	1.9	1.9	1.2	0.7	0.6
3.89	C_11_H_22_O	Undecanal	170.17	902	ND	ND	ND	ND	ND	ND	ND	ND	ND	ND	0.2	0.4	0.2	0.2	1.1	0.5	0.9	0.8
4.65	C_11_H_24_	Undecane	156.19	1,100	0.1	0.2	0.2	0.2	0.1	0.1	ND	ND	ND	0.0	ND	ND	0.1	ND	ND	ND	ND	ND
5.56	C_23_H_48_O	Tricosanol	340.37	743	0.3	0.3	0.5	0.4	0.4	0.2	0.1	0.4	0.1	0.6	ND	ND	0.3	ND	ND	ND	ND	ND
5.56	C_12_H_14_O_2_	trans-Ligustilide	190.10	825	ND	ND	ND	ND	ND	ND	ND	ND	ND	ND	0.8	0.3	0.3	0.3	0.6	0.4	0.2	0.2
5.87	C_17_H_36_O	1-Hexadecanol, 2-methyl-	256.28	816	0.7	0.6	0.9	0.9	0.6	0.4	0.2	0.3	0.1	0.4	ND	ND	0.5	ND	ND	ND	ND	ND
6.10	C_14_H_28_O_2_	Tetradecanoic acid	228.21	1768	0.5	0.6	0.3	0.5	0.2	0.6	0.6	0.5	0.7	1.0	0.6	0.7	0.4	0.6	0.8	0.5	0.9	0.9
6.19	C_16_H_34_O	2-Hexyldecanol	242.26	824	0.4	0.3	0.3	0.6	0.2	ND	ND	ND	ND	ND	ND	ND	0.2	ND	ND	ND	ND	ND
7.76	C_15_H_30_O_2_	Pentadecanoic acid	242.22	1867	1.4	1.5	1.1	1.6	0.8	1.3	1.3	1.1	1.7	1.1	ND	ND	0.7	ND	ND	ND	ND	ND
8.09	C_16_H_34_	Hexadecane	226.27	1,411	0.2	0.1	0.2	0.2	0.1	0.1	0.0	0.0	0.0	ND	ND	ND	0.1	ND	ND	ND	ND	ND
8.71	C_17_H_34_O_2_	Hexadecanoic acid, methyl ester	270.26	1,468	0.2	0.2	0.3	0.5	0.2	0.3	0.6	0.6	0.9	0.4	0.2	0.3	0.5	0.3	0.2	0.2	0.2	0.2
9.23	C_17_H_24_O	Falcarinol	244.18	1997	1.7	0.8	2.2	0.7	1.1	2.2	ND	1.7	ND	1.8	ND	ND	ND	ND	ND	ND	ND	ND
9.30	C_16_H_30_O_2_	9-Hexadecenoic acid	254.22	844	ND	ND	ND	ND	ND	ND	ND	ND	ND	ND	0.5	0.6	0.5	0.5	0.8	0.5	0.9	ND
9.82	C_16_H_32_O_2_	Palmitic acid	256.24	1968	29.4	28.0	23.6	ND	0.0	29.0	34.2	20.2	38.2	26.2	10.3	15.1	0.0	10.7	8.3	8.1	9.2	ND
11.80	C_18_H_24_O_2_	Methyl 5,8,11-heptadecatriynoate	272.18	766	0.7	0.8	1.2	0.7	0.3	1.6	0.7	1.7	1.2	0.9	ND	ND	0.3	ND	ND	ND	ND	ND
12.22	C_19_H_34_O_2_	8,11-Octadecadienoic acid, methyl ester	294.26	1940	ND	ND	ND	ND	ND	ND	ND	ND	ND	ND	0.7	1.2	1.4	0.9	0.5	0.6	1.0	0.9
13.73	C_18_H_32_O_2_	Linoleic acid	280.24	2,133	11.5	13.3	18.7	17.3	24.6	ND	16.0	ND	11.9	7.4	15.7	18.1	ND	14.5	10.3	15.7	12.0	0.7
13.91	C_18_H_34_O_2_	Oleic Acid	282.26	2,141	9.5	6.6	2.8	2.5	4.1	9.7	7.3	6.2	8.4	3.0	1.2	ND	24.6	1.1	1.8	1.5	2.6	0.2
14.45	C_18_H_36_O_2_	Octadecanoic acid	284.27	2002	5.5	5.5	3.9	4.7	3.0	4.4	3.8	3.4	5.0	ND	1.9	2.5	2.8	1.6	1.6	1.8	2.3	8.8
17.82	C_21_H_36_O_4_	α-Glyceryl linolenate	352.26	828	ND	ND	ND	ND	ND	ND	ND	ND	ND	ND	0.4	0.3	0.4	0.2	0.3	0.5	0.5	0.5
18.25	C_20_H_36_O_2_	Mandenol isomer	308.27	756	0.3	ND	ND	0.0	ND	ND	ND	ND	ND	0.0	ND	ND	0.0	ND	ND	ND	ND	ND
18.61	C_8_H_14_O_2_	β-Octalactone	142.10	760	ND	ND	ND	ND	ND	ND	ND	ND	ND	ND	0.1	0.1	0.1	0.3	0.3	ND	0.1	0.5
19.45	C_22_H_44_O_2_	Dodecanoic acid, decyl ester	340.33	927	ND	ND	ND	ND	ND	ND	ND	ND	ND	ND	1.9	ND	0.5	2.3	1.8	1.7	1.1	0.9
19.51	C_20_H_40_O_2_	Eicosanoic acid	312.30	2,362	1.1	1.3	ND	0.9	ND	0.9	0.7	0.9	1.1	0.7	ND	ND	0.5	ND	ND	ND	ND	ND
21.97	C_26_H_52_O_2_	Tetradecyl laurate	396.40	767	ND	ND	ND	ND	ND	ND	ND	ND	ND	ND	0.6	0.2	0.1	0.7	0.7	0.5	0.2	ND
24.05	C_16_H_26_O_3_	Dodecenyl succinic anhydride	266.19	826	ND	ND	ND	ND	ND	ND	ND	ND	ND	ND	6.7	2.8	2.1	7.3	5.6	8.2	5.8	5.9
24.45	C_24_H_48_O_2_	Dodecyl laurate	368.37	875	ND	ND	ND	ND	ND	ND	ND	ND	ND	ND	1.0	0.4	0.2	1.3	2.1	1.4	0.6	ND
24.60	C_22_H_44_O_2_	Docosanoic acid	340.33	2,566	1.9	3.0	1.6	2.4	0.8	2.1	1.8	1.3	2.2	0.7	ND	ND	0.6	ND	ND	ND	ND	ND
27.02	C_23_H_46_O_2_	Tricosanoic acid	354.35	758	0.4	0.8	0.3	0.5	0.1	0.4	0.5	0.2	0.5	0.1	ND	ND	ND	ND	ND	ND	ND	ND
29.27	C_26_H_52_O_2_	Hexadecanoic acid, decyl ester	396.40	938	ND	ND	ND	ND	ND	ND	ND	ND	ND	ND	2.5	0.9	0.8	2.9	1.7	2.3	2.2	ND
29.41	C_24_H_48_O_2_	Tetracosanoic acid	368.37	764	0.3	0.7	0.2	0.6	0.0	0.5	0.5	0.1	0.5	0.1	ND	ND	0.0	ND	ND	ND	ND	ND
31.52	C_18_H_24_O	3-Deoxyestradiol	256.40	789	ND	ND	ND	ND	ND	ND	ND	ND	ND	ND	0.7	0.3	0.2	0.9	0.7	ND	0.5	ND
33.07	C_20_H_36_O_2_	Mandenol	308.27	756	ND	ND	ND	ND	ND	ND	ND	ND	ND	ND	8.7	1.1	4.7	9.6	1.7	9.2	12.6	12.5
33.71	C_34_H_68_O_2_	Octadecyl palmitate	508.52	807	ND	ND	ND	ND	ND	ND	ND	ND	ND	ND	1.7	0.8	0.6	1.9	0.0	2.0	1.7	2.2
35.21	C_22_H_40_O_2_	Butyl 9,12-octadecadienoate	336.30	896	ND	ND	ND	ND	ND	ND	ND	ND	ND	ND	2.2	1.1	0.9	9.6	1.7	9.2	12.6	12.5
37.32	C_21_H_38_O_2_	Isopropyl linoleate	322.29	864	ND	ND	ND	ND	ND	ND	ND	ND	ND	ND	5.8	1.1	3.9	9.6	1.7	9.2	12.6	12.5
38.28	C_30_H_50_O	Stigmasterol methyl ether	426.39	829	0.3	0.4	0.3	0.6	0.7	0.2	0.2	0.6	0.2	0.4	ND	ND	0.5	ND	ND	ND	ND	ND
38.48	C_37_H_76_O	1-Heptatriacotanol	536.59	855	0.7	1.3	0.6	0.8	0.3	0.7	1.0	0.4	0.7	0.6	0.9	0.4	0.0	0.6	0.5	0.6	ND	1.0
39.37	C_29_H_48_O	Stigmasterol	412.37	3,170	4.1	6.7	3.4	5.9	2.2	0.0	5.2	2.9	3.7	2.5	0.7	1.0	ND	0.6	0.5	0.6	0.6	0.9
40.60	C_29_H_50_O	β-Sitosterol	414.39	3,200	1.1	1.3	0.9	1.1	0.9	0.7	0.7	0.7	0.7	0.6	ND	ND	0.4	ND	ND	ND	ND	ND

Note: 1–10, BCH001-010; 11–18, NCH, 001–008; ND, not detected.

According to classification based on the types of chemical structure (Figure 3B), majorities of SFEs components in BCH and NCH belong to fatty acids (averaged relative contents of 51.07 and 50.36%), sterols (averaged relative contents of 4.53 and 1.06%) and, miscellaneous components (averaged relative contents of 2.86 and 9.98%). Nevertheless, the compositions and relative contents of the major components in SFEs of BCH and NCH varied greatly between these two species. As illustrated in Figure 3C, lactones, aldehydes, acetates and fatty alcohols were much more abundant in NCH, while BCH contained more sterols by contrast. These results further indicated the significant differences in chemical types of the major components between BCH and NCH.

### Species discrimination of BCH and NCH with metabolomic-based chemometrics

**FIGURE 4:**
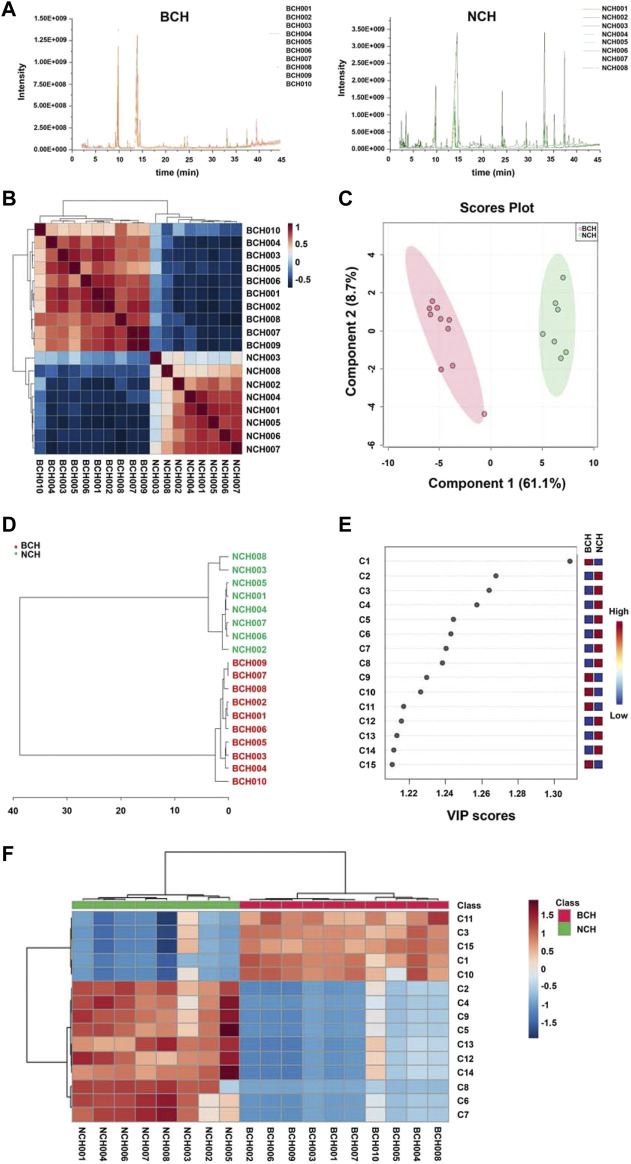
Chemometric analysis for species discrimination of BCH and NCH. **(A)** The GC-MS chromatograms of BCH (BCH001-010) and NCH (NCH001-008); **(B)** the SRCCA visualization for the discrepancy between BCH and NCH; **(C)** the PLS-DA analysis of BCH and NCH; **(D)** the HCA discrimination of BCH and NCH; **(E)** the top 15 features ranked by VIP values; and **(F)** Hierarchical clustering Heatmap of top 15 features ranked by *t*-test value. The colored boxes on the right indicated the relative concentrations of the corresponding metabolite in each group under study. **C1**, tricosanoic acid; **C2**, dodecenyl succinic anhydride; **C3**, dodecanoic acid; **C4**, lauryl acetate; **C5**, 1-dodecanol; **C6**, mandenol; **C7**, isopropyl linoleate; **C8**, octadecyl palmitate; **C9**, docosanoic acid; **C10**, tetracosanoic acid; **C11**, methyl 5,8,11-heptadecatriynoate; **C12**, undecanoic acid; **C13**, tridecane; **C14**, trans-3-octadecene; and **C15**, β-sitosterol.

Chemometric analysis including SRCCA, PLS-DA and HCA were employed to visualize the discrepancy and screen the potential makers for species discrimination of BCH and NCH. The GC-MS chromatograms of all BCH (BCH001-010) and NCH (NCH001-008) samples were illustrated in Figure 4A. Prior to statistical analysis, features with low repeatability in all samples were removed by setting the value of relative standard deviation (RSD) at 25%. Moreover, data of all samples were normalized by sum then were transformed to a binary logarithm (log^2^ *X*, where *X* represents the peak area) to avoid the instrument error.

As illustrated in Figure 4B, the results of SRCCA indicated a significant low inter-species correlation while strong intra-species correlation between these two species. The cross validation [R^2^Y (cum) = 0.93, Q^2^ (cum) = 0.90] also suggested the good predictive capability and the significant explanatory power of PLS-DA model for effective species discrimination of BCH and NCH (Figure 4C). In addition, the grouping trend was confirmed by HCA, which could be seen from the well clustered groups of BCH and NCH (Figure 4D). All these results further suggested the significant difference in chemical compositions between BCH and NCH.

VIP values and *t*-test were used to screen of potential makers for differentiation of BCH and NCH. As shown in Figures 4E,F, 15 markers with higher VIP value or intensity change evaluated with *t*-test were selected, including tricosanoic acid (**C1**), dodecenyl succinic anhydride (**C2**), dodecanoic acid (**C3**), lauryl acetate (**C4**), 1-dodecanol (**C5**), mandenol (**C6**), isopropyl linoleate (**C7**), octadecyl palmitate (**C8**), docosanoic acid (**C9**), tetracosanoic acid (**C10**), methyl 5,8,11-heptadecatriynoate (**C11**), undecanoic acid (**C12**), tridecane (**C13**), trans-3-octadecene (**C14**) and β-sitosterol (**C15**). Among these components, **C1** and **C11** exclusively existed in BCH, and **C4**, **C5**, **C9**, **C12**, **C13** and **C14** were only detected in NCH. Moreover, the contents of **C2**, **C6**, **C7** and **C8** in NCH were significantly higher in NCH than those in BCH.

**FIGURE 5:**
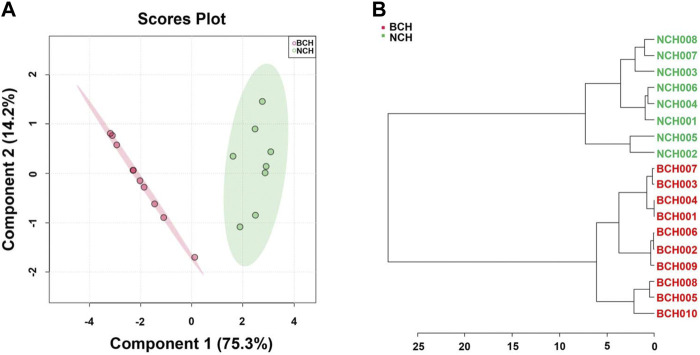
Accuracy evaluation of potential marker compounds. **(A)** BCH and NCH were well discriminated at specie level by PLS-DA using 15 optimized potential makers; **(B)** BCH and NCH were well separated by HCA using 15 optimized potential makers.

To further verify the accuracy and feasibility of the optimized combination of 15 chemical components for specie discrimination of BCH and NCH, the relative content of above 15 markers were normalized to build new Chemometric models. As illustrated in Figure 5, individual BCH and NCH species could be well discriminated under multiple chemometric models (including PLS-DA, HCA) with above 15 markers, and the new models presented a comparably satisfied performance for discrimination of two BR species (R^2^Y (cum) = 0.940, Q^2^ (cum) = 0.891).

## Discussion

Due to the complex sources of Bupleurum plants, the herbal materials of Bupleuri Radix (BR) are chaotic in clinical applications. Although only the plants of *B. chinense* DC. (BCH) and *B. scorzonerifolium* Willd. (NCH) are officially authenticated to be used for decoction pieces of BR, the difference in chemical compositions between them could be seen from the previous publications of chemical analysis for individual species (Yang et al., 2010; Tykheev et al., 2018). However, the comparative investigation on the similarity and difference between BCH and NCH is still not reported. Therefore, in this study, the chemical compositions of the anti-inflammatory extracts from BCH and NCH were firstly comprehensively compared for further selection of the potential chemical markers for species discrimination.

As reported by previous publications, the volatile components are one of the most important parts of chemicals in BR (Meng et al., 2014; Li et al., 2015) and associated to its pharmacological activities for antipyretic and anti-inflammation (Ni et al., 2009; Chen et al., 2010). Considering the low contents of essential oils (HDEs) in the BR (yield of 0.1 and 0.25% for BCH and NCH by hydro-distillation extraction in this study), the extracts of BCH (average yield: 1.16 ± 0.06%) and NCH (average yield: 3.57 ± 0.44%) prepared by supercritical fluid extraction (SFEs) were also involved for comparison. Beside, as summarized in Table 2, compounds including palmitic acid, linoleic acid, octadecanoic acid, 1-dodecanol, lauryl acetate, mandenol and dodecenyl succinic anhydride were common components of NCH independence on habitats; while falcarinol, palmitic acid, linoleic acid, oleic acid, octadecanoic acid, eicosanoic acid and docosanoic acid were consistent compounds of BCH independence on habitats. However, their content varied from traces to significant amounts in individual specie, and some components were not detected in samples from other regions, suggesting certain difference in intra-species for BCH and NCH from different regions. As shown in Figures 4B,D, these intra-species differences allow samples divided into groups almost according to the geographical regions, indicating habitats of individual specie also contributed to the chemical differences.

Apart from the higher potent of the NCH than the BCH, the SFEs (IC_50_ of 6.39 ± 0.52 and 1.32 ± 0.05 mg (herb)/mL for BCH and NCH) were observed to present much better activities against LPS-stimulated inflammation (NO release) in RAW264.7 macrophages than those of the HDEs (IC_50_ of 203.90 ± 8.08 and 32.32 ± 2.27 mg (herb)/mL for BCH and NCH). The obtained results suggested the contribution of some other hydrophobic components in BR to anti-inflammation.

The compositional analysis of the SFEs of BCH and NCH were preformed using the GC-MS system. BCH exhibited a significant difference to NCH in both the chemical constituents and the contents of the main components containing in the SFEs. A total of 48 components of different chemical types have been identified in the SFEs of BCH and NCH, including 24 common components as well as 4 and 20 specific components for BCH and NCH, respectively. Among them, lactones, aldehydes, acetates and fatty alcohols were significantly abundant in NCH, while sterols were more enriched in BCH. The characteristic distribution of the volatile (aromatic) components reveals the different smell for each decoction pieces as specifically described in the Chinese pharmacopeia for BCH (aroma) and NCH (transmutative-oil-like).

Chromatography-based untargeted metabolomics analyses are rapid and reliable approaches which have been quickly developed and applied for the quality research of Chinese herbal medicines in recent years (Tao et al., 2018; Lu, et al., 2022; Xiao, et al., 2022). However, appropriate screening and selection of the chemical markers with minimum combination for effective species discrimination is very important for such studies. In this study, by combining various chemometric models (HCA, PLS-DA, and SRCCA) as well as the VIP values and intensity changes evaluated by the *t*-test, 15 makers from the identified components in the SFEs of BCH and NCH were discovered. As shown in Figure 4F, the selected components could be divided into three groups including the non-specific components (**C2**, **C3**, **C6**, **C7, C8 and C10**), BCH-specific components (**C1** and **C11**) and NCH-specific components (**C4**, **C5**, **C9**, **C12**, **C13** and **C14**). Among the non-specific components, the contents of **C2**, **C6**, **C7** and **C8** in NCH were significantly higher in NCH than those in BCH. These higher-content and specific components in NCH might be mainly responsible for its significantly potency on anti-inflammation. Furthermore, the good species discrimination with the optimized components combination was verified under PLS-DA and hierarchical cluster analyses. The results suggested the potential selection of the chemical markers from this combination for the quality control of BCH and NCH.

## Conclusion

In summary, the similarity and difference between two BR plants including *B. chinenses* DC (BCH) and *B. scorzonerifolium* Willd (NCH) were firstly comprehensively compared and reported both in chemistry and pharmacology. The higher anti-inflammatory activities of the SFEs than the HDEs suggested the involvement of some hydrophobic components apart from the essential oils on the functions of BR against fever and inflammation. Moreover, the developed minimum chemical combination including 15 components from both BCH and NCH was successfully applied for the discrimination of individual species. The contribution of this study is not only help us to better understand the activity related chemical difference between BCH and NCH, but also provide the potential selection of chemical markers for further improvement of the quality control standard for BCH and NCH.

## Data Availability

The original contributions presented in the study are included in the article/supplementary material, further inquiries can be directed to the corresponding authors.

## Abbreviations

BCH, beichaihu; BR, bupleuri Radix; GC-MS, gas chromatography-mass spectrometry; HCA, hierarchical clustering analysis; HDEs, hydro-distillation extracts; IC50, 50% inhibitory concentration; LPS, lipopolysaccharides; NCH, nanchaihu; NO, nitric oxide; PLS-DA, Partial least square discrimination analysis; SFEs, supercritical fluid extracts.
